# Investigating the influence of institutions, politics, organizations, and governance on the COVID-19 response in British Columbia, Canada: a jurisdictional case study protocol

**DOI:** 10.1186/s12961-022-00868-5

**Published:** 2022-06-21

**Authors:** Laura Jane Brubacher, Md. Zabir Hasan, Veena Sriram, Shelly Keidar, Austin Wu, Michael Cheng, Chris Y. Lovato, U. B. C. Working Group on Health Systems Response to COVID-19, Peter Berman

**Affiliations:** School of Population and Public Health, University of British Columbia, 2206 East Mall, Vancouver, BC V6T 1Z3 Canada

**Keywords:** COVID-19, Case study, Institutions, Politics, Organizational structure, Governance, Public health, Health crisis response, Preparedness, Lessons learned

## Abstract

**Background:**

Research on public health responses to COVID-19 globally has largely focused on understanding the virus’ epidemiology, identifying interventions to curb transmission, and assessing the impact of interventions on outcomes. Only recently have studies begun to situate their findings within the institutional, political, or organizational contexts of jurisdictions. Within British Columbia (BC), Canada, the COVID-19 response in early 2020 was deemed highly coordinated and effective overall; however, little is understood as to how these upstream factors influenced policy decisions.

**Methods:**

Using a conceptual framework we developed, we are conducting a multidisciplinary jurisdictional case study to explore the influence of institutional (I), political (P), organizational (O), and governance (G) factors on BC’s COVID-19 public health response in 2020–2021. A document review (e.g. policy documents, media reports) is being used to (1) characterize relevant institutional and political factors in BC, (2) identify key policy decisions in BC’s epidemic progression, (3) create an organizational map of BC’s public health system structure, and (4) identify key informants for interviews. Quantitative data (e.g. COVID-19 case, hospitalization, death counts) from publicly accessible sources will be used to construct BC’s epidemic curve. Key informant interviews (*n* = 15–20) will explore governance processes in the COVID-19 response and triangulate data from prior procedures. Qualitative data will be analysed using a hybrid deductive–inductive coding approach and framework analysis. By integrating all of the data streams, our aim is to explore decision-making processes, identify how IPOG factors influenced policy decisions, and underscore implications for decision-making in public health crises in the BC context and elsewhere. Knowledge users within the jurisdiction will be consulted to construct recommendations for future planning and preparedness.

**Discussion:**

As the COVID-19 pandemic evolves, governments have initiated retrospective examinations of their policies to identify lessons learned. Our conceptual framework articulates how interrelations between IPOG contextual factors might be applied to such analysis. Through this jurisdictional case study, we aim to contribute findings to strengthen governmental responses and improve preparedness for future health crises. This protocol can be adapted to and applied in other jurisdictions, across subnational jurisdictions, and internationally.

**Supplementary Information:**

The online version contains supplementary material available at 10.1186/s12961-022-00868-5.

## Background

Throughout 2020–2021, research on public health responses to the COVID-19 pandemic globally largely focused on understanding the virus’s epidemiology [1–3], identifying clinical interventions as well as public health and social measures to curb transmission [4, 5], and assessing the impact of interventions on outcomes [6, 7]. Despite vast jurisdictional differences in COVID-19 outcomes, and the process and extent to which a similar suite of interventions were implemented, few studies have situated their findings within institutional, political, governance, or organizational contexts, broadly representing upstream determinants of the COVID-19 response. A growing body of work is exploring the influence of these factors on public health crisis response and pandemic preparedness, recognizing their influence on variability of responses across jurisdictions and how they might underscore key lessons learned for future responses [8–10].

For instance, the level of trust in government [8, 11], (de)centralization of state authority [9, 12], protection of democratic principles [13, 14], degree of political partisanship [14, 15], subnational politics [16], and activation of intra- and intercity organizational networks [17] reportedly influenced COVID-19 governmental response, and by extension disease transmission, within jurisdictions. However, a consolidated, interdisciplinary framework taking a holistic view of upstream determinants of the public health response to COVID-19 has been a key gap. While previous research indicates the importance of institutions, politics, organizational structures, and governance to public health responses, a gap exists in research that explores the dynamic interrelationships *between* these factors in the context of pandemic response and—further—defines, describes, and characterizes their influences in depth. This inquiry is critical for understanding the origin, nature of, and rationale for government decisions and actions and, thus, strengthening capacity to respond to future public health crises [10].

Within British Columbia (BC), Canada, the COVID-19 response in early 2020 was widely perceived by the public and media to be highly coordinated and effective overall [18–21]; however, little is understood as to how these upstream contexts in which public health decision-making occurs influenced policy decisions and implementation, and contributed to the success of this response, or how this response evolved following the “first wave” of infections in early 2020. As such, we aim to conduct a jurisdictional case study to explore the influence of these upstream factors on BC’s COVID-19 public health decision-making. Our specific objectives are to (1) describe and characterize the potential influence of institutions, politics, organizations, and governance (IPOG) on BC’s COVID-19 public health response in 2020–2021, and (2) identify lessons learned and best practices for public health emergency response, from the perspectives of BC stakeholders. By expanding understanding of the ways in which IPOG factors interactively influenced decision-making, from the perspectives of those involved, this study may illuminate implications for strengthening governmental response to future public health crises across various types of jurisdictions.

To guide this work we have developed a conceptual framework, situating institutions (I), politics (P), organizational structures (O), and governance (G)—here termed IPOG—within the broader societal context and assuming that the dynamic interplay between these factors will help explain public health decision-making. A recent scoping review of existing public health frameworks for evaluation of epidemic responses characterized elements of an effective epidemic response into five central “threads of analyses”, including context, intervention, process, performance, and impact analyses [22]. Of these, “context analysis” had the fewest existing public health frameworks associated with it. Our IPOG conceptual framework, developed for use in this proposed study, articulates how interrelations between various upstream components (e.g. the concepts of I, P, O, and G) might be applied to analysis of public health crisis response and management including circumstances beyond the current COVID-19 crisis.

The continued response to the COVID-19 crisis is already stimulating deeper reviews of public health capacities and response as part of preparedness for future crises [23, 24]. The work presented here can contribute to analysis of how IPOG factors influenced emergency responses in different jurisdictions. Ultimately, the purpose of this work would be to generate more systematic learnings of how laws, regulations, and organizational design can help support more effective preparation and response to improve outcomes and equity in the future.

## Methods

### IPOG: a conceptual framework

**Fig. 1:**
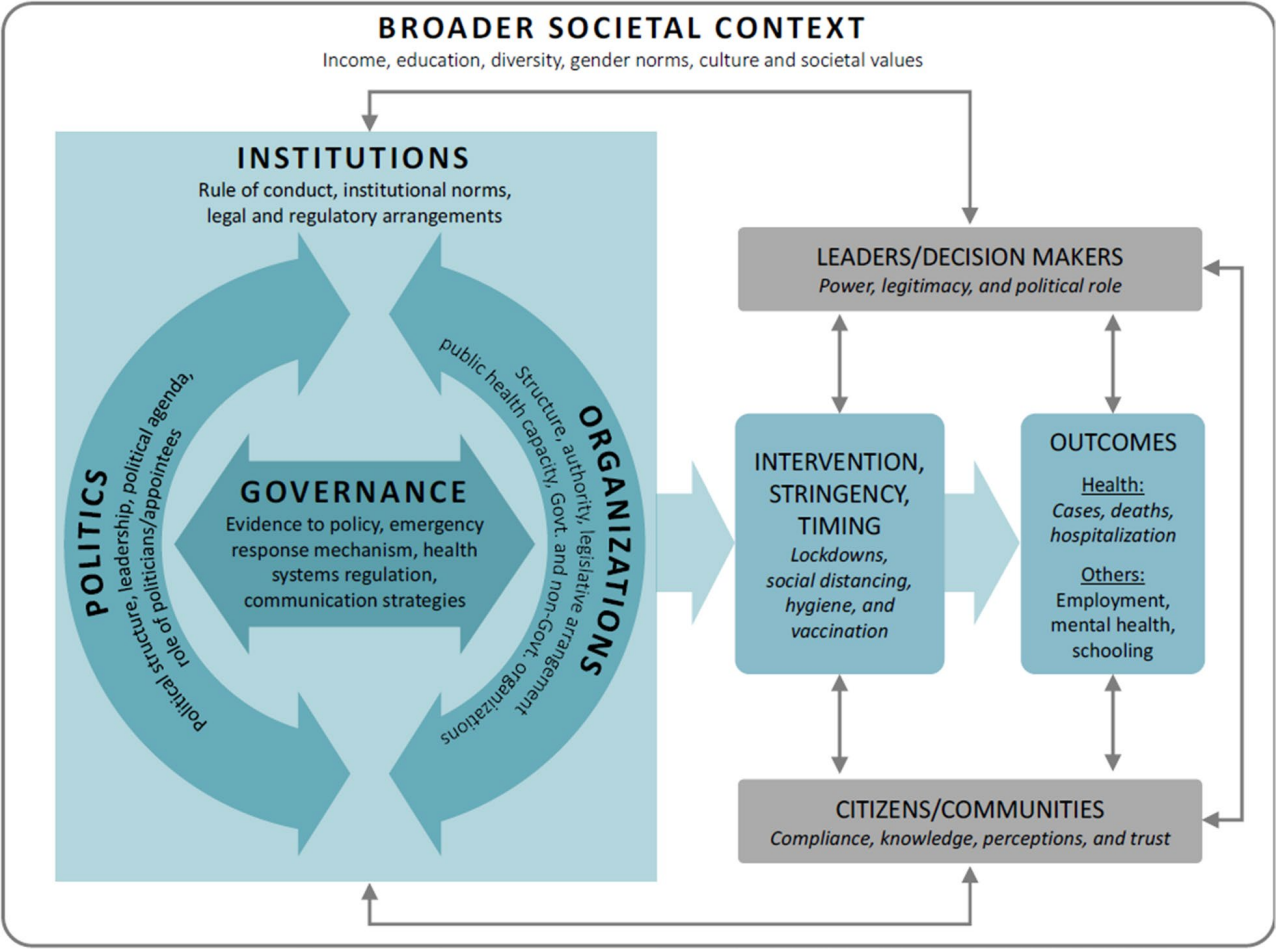
The IPOG conceptual framework was developed for this proposed study

We developed a conceptual framework that situates institutions, politics, organizations, and governance in relation to one another and provides an analytical lens through which to explore the public health pandemic response (Fig. 1).

Within this framework, the complex term “governance” (G) is focused on processes of decision-making at the interface between political (P) and organizational structures (O) [25], all of which is conditioned by institutions (I) (e.g. norms of behaviour and rules of conduct [26, 27]). Leaders, as well as individual citizens and communities, interact with this IPOG locus. Broader societal influences—social determinants of health such as income and education levels, as well as other cultural and societal norms and values—provide important context, as they also shape and define IPOG in a given jurisdiction, along with the roles and perspectives of leaders and citizens. I, P, O, and G have potential to influence the stringency and timing of public health interventions and, thus, the resulting outcomes. Both interventions and outcomes impact leaders and citizens, feeding back into the I, P, O, and G structures and processes. Further details on how we have defined and operationalized the elements of this conceptual framework are provided elsewhere [28] (see Additional file 1).

### Overall study design

**Fig. 2:**
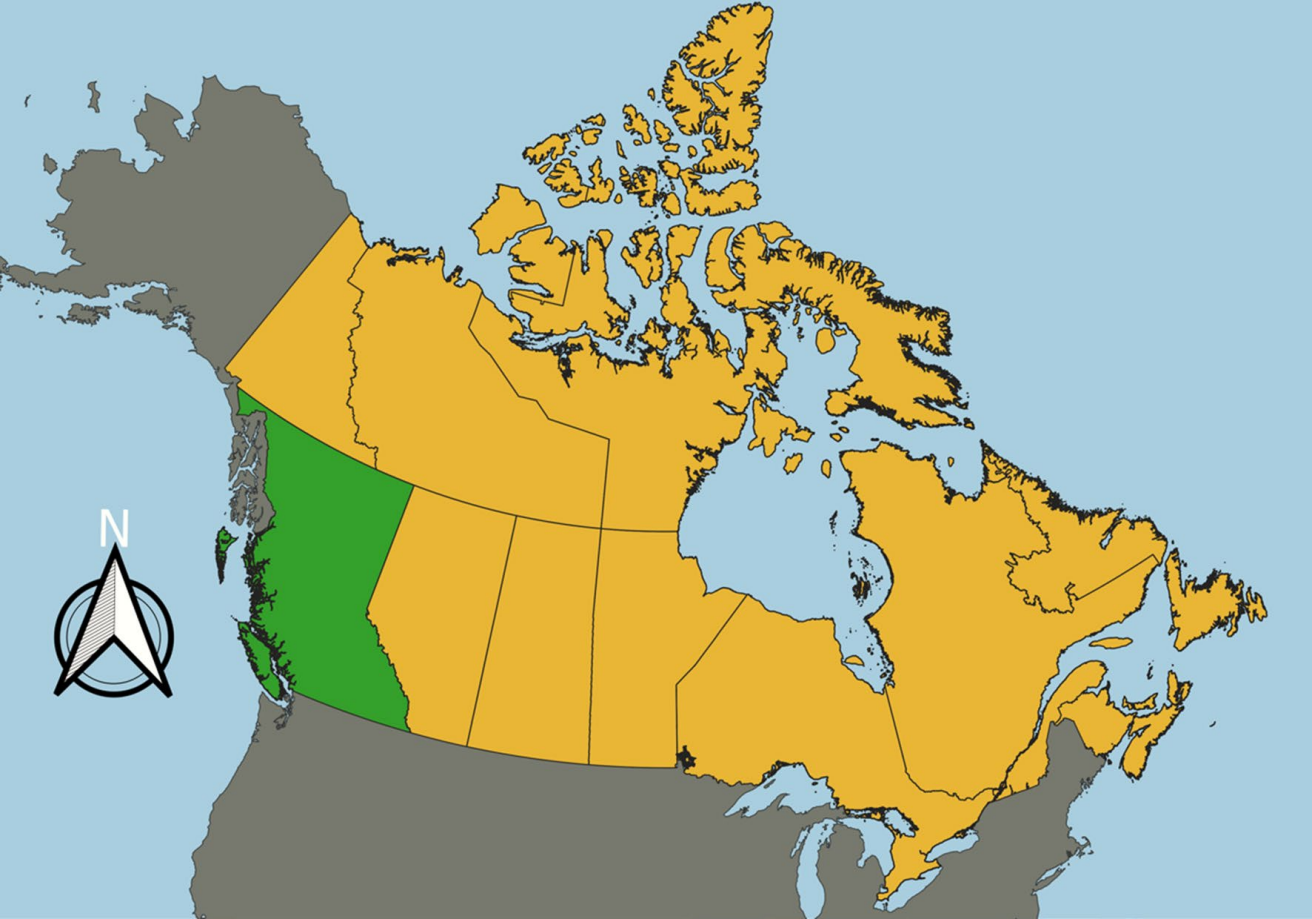
Map of Canada (yellow shading), indicating the westernmost province of BC (green shading)

**Fig. 3:**
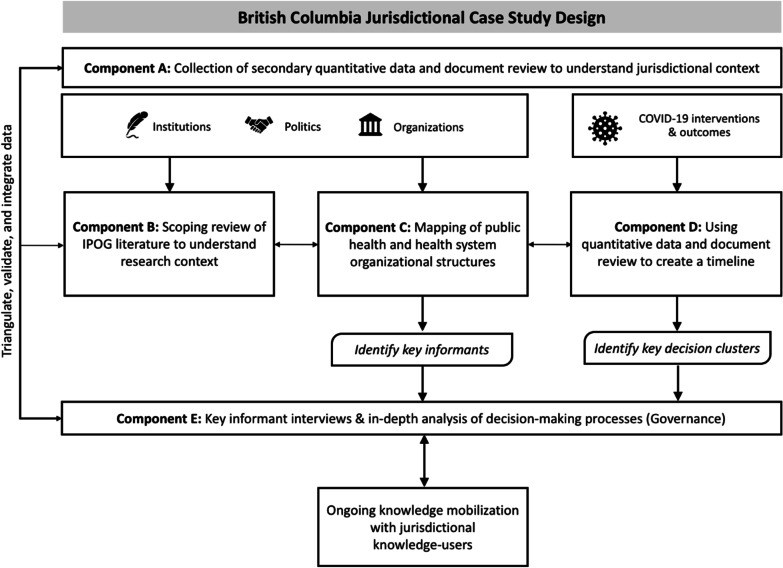
Overall jurisdictional case study design

Our case study will be conducted within BC, a province of 5.21 million people in western Canada (Fig. 2), guided by Yin’s (2009) single-case study design [29]. This approach is characterized by the use of multiple data sources and data collection procedures for triangulation, to generate a more comprehensive understanding of a phenomenon (Fig. 3) [30]. In this case, both quantitative and qualitative data will be used to explore factors and processes that influenced the government’s decision-making. Overall, this jurisdictional case study approach will also generate data associated with specific time periods in the COVID-19 response, and how IPOG factors influenced processes of decision-making across the pandemic progression in 2020–2021.

This proposed jurisdictional case study has received ethics approval through the University of British Columbia’s Institutional Research Ethics Board (Certificate #: H20-02136). The overall study design and specific methods proposed were informed by a series of virtual roundtable discussions we hosted with multidisciplinary scholars (e.g. from epidemiology, political science) and non-academic practitioners [31], as well as by ongoing collaboration with international research partners conducting similar studies in their jurisdictions.

### Case study components: data collection and analytical approaches

For each concurrent data collection and/or analytical approach noted below, additional supplementary material is provided in Appendices B–E.

#### Component A: Understanding the jurisdictional context—gathering secondary data and conducting a document review

We will gather and synthesize publicly available data as a foundation for understanding the BC jurisdictional context. This includes national and provincial data on a broader social context, such as population demographics, socioeconomic data, and geographic data available in the public domain and in relevant grey literature (e.g. research papers and reports).

Additionally, a document review will be conducted to gather and organize relevant documents from the public domain pertaining to, for instance, COVID-19 policy decisions, and public health and social measures implemented [32]. These relevant policy documents and media reports may be used to characterize institutional and political factors relevant to BC’s COVID-19 response and to identify key informants for interviews. Through this process, we may note important institution-related factors in our jurisdictional context, for example, compliance to the rule of law, relevant beliefs about individual and social responsibilities﻿, and trust in evidence and science [33]. Review of the political manifestos of ruling and opposition parties, tenure of key elected officials or others in positions of authority, and the roles of political appointees in government bureaucracy may contribute to our description of political factors influencing the response [34].

#### Component B: Scoping review of the literature

A scoping review of peer-reviewed and grey literature will be conducted to explore how the concepts of I, P, O, and G have been understood and operationalized in relation to the literature on public health crisis response and preparedness. This review will also characterize the extent, range, and nature of global IPOG-related literature as it relates to public health crisis response, to provide the context within which to understand our case study and contribution to current and future research (see Additional file 1: File 2).

#### Component C: Mapping of public health and health system organizational structures

**Fig. 4:**
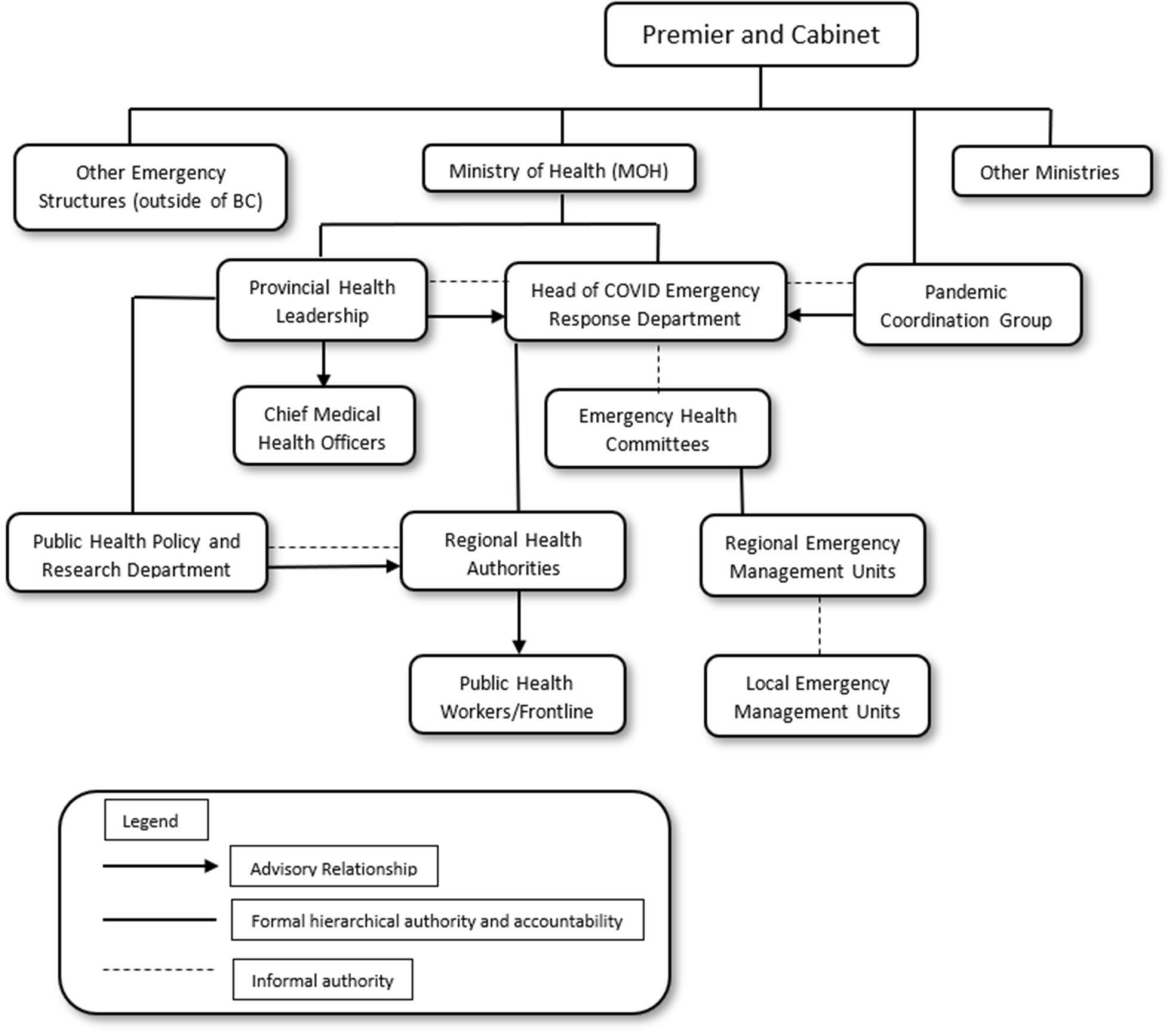
Example of an organizational map of a public health system, indicating formal and informal relationships between entities/roles (e.g. denoted in law or regulations), reporting hierarchy, and accountability

We aim to construct a visual map, or organogram [35], of the BC public health and health system organizational structure and functions to understand the key organizational actors involved, relationships of accountability and channels of communication between them, and the loci of processes involved in the COVID-19 response. By mapping the formal structures and relationships of key BC organizations (governmental and others) that determine and implement public health interventions, we will also identify key informants to interview. We will map both the “normative” or “de jure” relationships outlined in publicly available sources such as laws, regulations, formal standard operating procedures, and government websites, as well as informal relationships of influence, reporting, and accountability discussed by key informants in interviews (Fig. 4) [component E] [35, 36]. Since the organization of systems may change in an emergency, we will create both pre-COVID-19 and during-COVID-19 organizational maps, and iterate as more data are collected (see Additional file 1: File 3).

#### Component D: Creating an epidemic curve and timeline of associated “decision clusters” in the BC government’s response

Descriptive quantitative data on BC’s epidemic progression (e.g. case, hospitalization, and death counts; other relevant COVID-19 outcomes) will be collected from publicly accessible data sources. These include WHO, Johns Hopkins University, or Oxford University (global databases), and the Canadian Institute for Health Information (national database). Using these data, we will construct a series of epidemic curves for BC to visualize COVID-19 outcomes over time from early 2020 to the end of 2021.

**Fig. 5:**
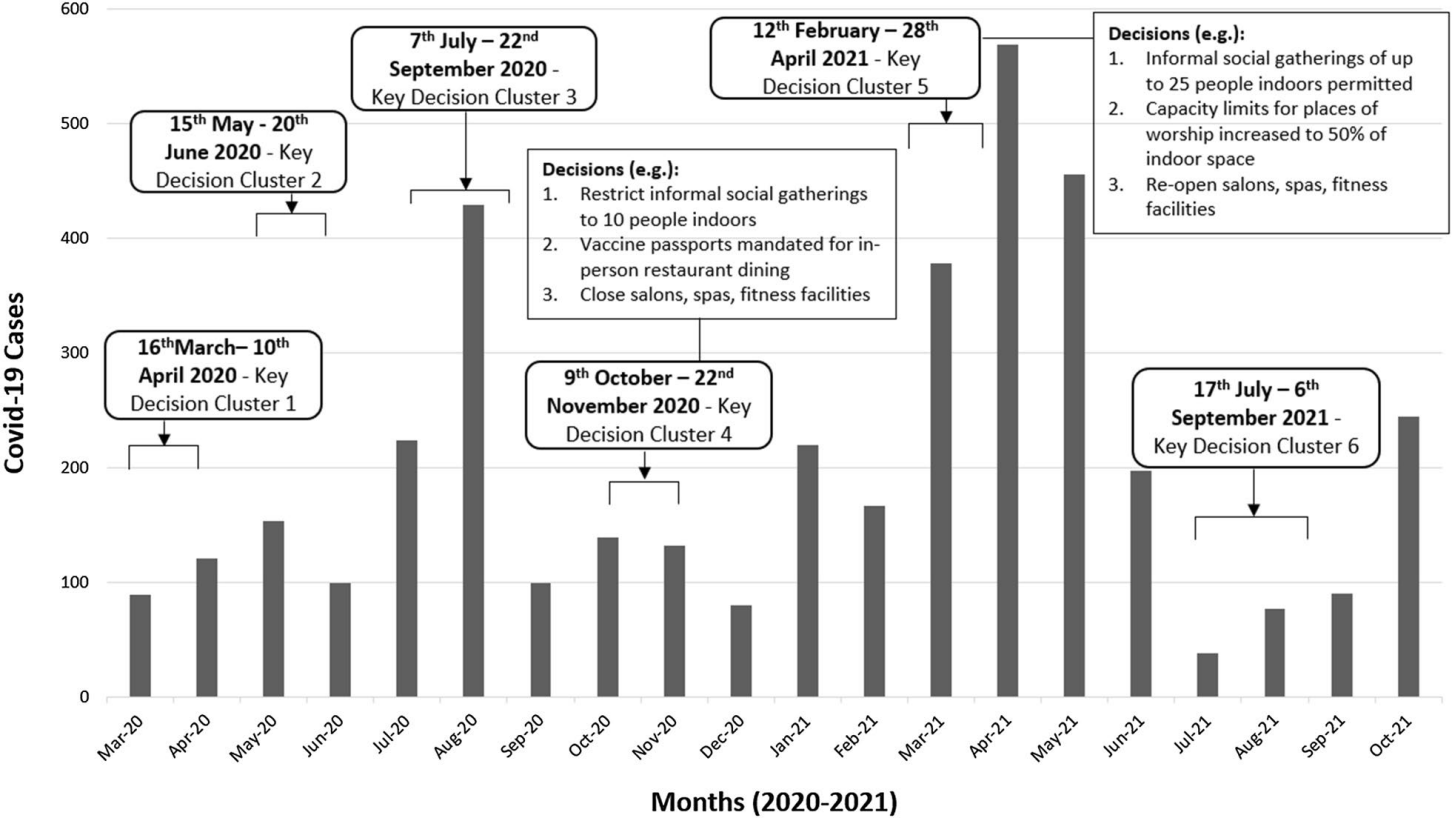
Example of an epidemic curve and associated timeline of decision clusters plotted chronologically on the same axes

Concurrently, we will create a chronological database of BC government decisions throughout the COVID-19 response, drawing data from relevant media reports, government reports, and policy documents retrieved in the document review [component A]. We aim to identify key clusters of decisions^1^ in the epidemic progression and plot these as a timeline overlay on the epidemic curves (Fig. 5) (see Additional file 1: File 4).

#### Component E: Exploring governance processes in BC’s COVID-19 response through key informant interviews

We aim to conduct approximately 15–20 semi-structured interviews with key informants who participated in, or are knowledgeable about, decision-making processes in BC’s pandemic response [37]. These respondents are likely those embedded within the formal political and organizational structures mapped in our organogram [component C], as well as non-state actors such as representatives of key interest groups. Thus, our organizational mapping procedure will be used to identify and purposively sample potential interviewees. Snowball sampling will also be utilized, as interviewees will be asked to recommend other key informants involved in BC’s response.

Interviews will focus on the governance processes involved in decisions and how institutions, politics, and organizational structures and dynamics may have influenced the choice, timing, and stringency of interventions in the COVID-19 response. Sample questions (Box 1) will be reordered and adapted according to each interviewee’s background and involvement in epidemic response (see Additional file 1: File 5 for the full semi-structured interview guide). We anticipate that questions may be added to the interview guide based on findings from components A–D; for instance, informants may be asked to clarify reporting relationships between organizational units mapped in our organogram [component C] or to comment specifically on key decision clusters identified in generating our timeline [component D]. Interviews will be audio-recorded, with permission, and subsequently transcribed in full. All key informants will be asked to provide informed written consent prior to the interview.

**Table:** 

Box 1: Excerpt from semi-structured interview guide	
• Please describe your professional background and titles/positions (in which organizations) relevant to the COVID-19 pandemic response in the period leading up to 18 March 2020 • Were you personally involved in discussions about when and how to declare a provincial state of emergency in BC (18 March 2020)? In what ways were you involved? • Who else was involved in these decision-making processes? What were their roles and positions? • Several specific orders were launched under the authority of the declaration. What was your role in relation to these orders? With whom did you work or collaborate? What influenced those decisions? • In your engagement or contributions during that time, were you directly meeting with or communicating with persons holding political office, such as elected officials? Please describe some examples	

Data from key informant interviews will be used to both validate and fill any identified knowledge gaps in the timeline and organizational mapping procedures. As interviewees will be asked to identify and characterize decision clusters they consider pivotal for the overall pandemic response, as well as to describe their organizational roles and responsibilities in the response, these data will also provide a robust narrative to supplement and further understand the organizational mapping and timeline [components C and D, respectively].

In-depth qualitative analysis will be conducted concurrently with key informant interviews. Thematic analysis, using a hybrid deductive–inductive coding approach [38], will be used to generate analytical insights (with the deductive coding informed by our conceptual framework in Fig. 1). QSR NVivo software will be used for the organization and retrieval of codes and coded transcript excerpts. Framework analysis may also be utilized to help generate analytical insights across and within participant stakeholder groups (e.g. organizations) [39].

Our aim through this analysis will be to explore decision-making processes, with the goal of understanding constraints, facilitators, and other factors influencing decision clusters in BC’s epidemic progression, drawing out lessons for decision-making in public health crises, and potentially, developing theory on the effects of IPOG factors on public health response. This process will require the engagement of jurisdictional knowledge partners, who will be consulted for their feedback on what research recommendations and lessons learned are contextually relevant and useful for future planning and preparedness.

#### Integration and triangulation of findings; ongoing knowledge mobilization

Data and analytical insights generated through components A–E will be integrated iteratively and triangulated as the study progresses [40]. Mixed insights will highlight the relationships between IPOG factors and their relevance in relation to the epidemic progression in BC and public health decision-making. Throughout data collection and analyses, key identified knowledge users from within BC political and public health organizations will be engaged. Specifically, we will invite potential knowledge users to provide feedback on preliminary findings, implications, and recommendations for improving current and future responses to pandemics. Finalized results will be presented to knowledge users as a summary of key findings, lessons learned, and recommendations, in the form of a plain-language report; policy brief; and interactive knowledge-exchange session. Additional pathways for mobilizing findings for public health policy and practice will be identified as the research develops.

## Discussion

Increasingly, national governments and subnational jurisdictions are conducting retrospective examinations of the processes involved in their COVID-19 health policy decisions and actions to identify lessons learned [23, 24]. Using the case study protocol described in this paper, we aim to contribute findings that inform ongoing discussions of systems reform. Some of the lessons learned from this BC case study may be generalizable to other jurisdictions and useful for improving preparedness for and response to future health crises in Canada and internationally.

This protocol can be adapted to and applied in other jurisdictions. For instance, our research team is utilizing this IPOG approach for a comparative analysis of COVID-19 responses across subnational jurisdictions (e.g. Canadian provinces) and international jurisdictions with which we already have established research partnerships. This comparative approach may be used to identify common best practices across jurisdictions with respect to health crisis preparedness and response. Future studies might explore the influence of IPOG factors not only on decision-making processes, but also on the implementation and effectiveness of policy decisions.

This case study aims to respond to a critical research gap related to understanding and improving public health systems in Canada. The case study protocol described in this paper has potential for contributing to a critical knowledge gap related to understanding and improving the public health system [41]. While a whole-of-government response is needed in responding to public health threats, we must not overlook the influence of institutional, political, and organizational contexts in planning, implementing, and evaluating results. As such, the findings from this study, and others like it, will contribute to identifying the kind of changes needed to improve public health systems and, particularly, how they function in the context of a health crisis.

We anticipate some potential limitations in this study. For instance, interviews with senior decision-makers who are accountable to their organizations and to the public may provide responses that are largely influenced by political or organizational motivations. These informants may also be reluctant to openly discuss challenges and constraints to decision-making processes. In response, we will aim to recruit a diversity of key informants involved in varying aspects of pandemic decision-making, and will emphasize the confidentiality of each participant’s identity, as upheld in our study’s ethics protocol. Additionally, we may encounter challenges in accessing data on health system organizational structure and constituent relationships, for example, as these data are not always publicly available. Our approach of triangulating data from multiple data collection processes (e.g. organizational mapping and key informant interviews) may help to mitigate these challenges.

## Supplementary Information

**Additional file 1.** Further details on data collection and analytical approaches.

## Data Availability

Not applicable.

## Abbreviations

COVID-19: Coronavirus disease 2019
IPOG: Institutions (I), politics (P), organizations (O), governance (G) framework
BC: British Columbia, Canada

## Footnotes

1 We will define “decision clusters” as a series of decisions, as evidenced in government orders, rules, or statements, that are linked in purpose and proximate in time (e.g. increasing restrictions, relaxing restrictions, introducing new interventions).
