# Supplementary material for: Investigating the influence of institutions, politics, organizations, and governance on the COVID-19 response in British Columbia, Canada: a jurisdictional case study protocol
Source: Health Res Policy Syst. 2022 Jun 21;20:74. doi: 10.1186/s12961-022-00868-5 (PMC9210337; doi:10.1186/s12961-022-00868-5)
Supplement: Supplementary file 1 — Additional file 1. Further details on data collection and analytical approaches. [file 12961_2022_868_MOESM1_ESM.docx]

| **Additional file 1:** Working definitions of the Institutional, Political, Organizational, and Governance (IPOG) elements of the conceptual framework developed for this case study   \| **INSTITUTIONS**  Definition: Rules and enforcement characteristics that shape human interactions. Institutions structure incentives to which individuals and organizations respond. They can be formal or informal. Similar to the rules of a game, they give rise to meaningful practices and prescriptions for appropriate behavior among players.  *Example:*   - *Property rights and contracts; legal systems and their legitimacy, and adherence* - *Within health systems, institutions, for example, underpin the authority, respect, and confidence accorded to political leaders, public-health officers, and scientists,* - *Views of the legitimacy of interests of the private sector, general understanding of the balance between social and individual good* \| \| --- \| \| **POLITICS**  Definition: The arena in which political actors (organizations and individuals) are engaged in decision-making for policy, legislation, and management of the public sector. The political arena is structured by institutions that shape the roles and authority of decision-makers, including government, opposition, and civil society. Politics also exerts influence through the ways in which positions of elected and politically appointed officials engage with civil servants and technical experts.  *Example:*   - *Cabinets as an arena of high-level decisions and appointments, including politically appointed officials in government relative, senior civil servants, and experts* - *Electoral processes and legislatures as arenas of party competition and cooperation* - *Coalitions or agreements among parties* - *Interactions between political and bureaucratic actors contributing to policy decisions, directives, guidelines, orders related to COVID-19* \| \| **ORGANIZATIONS IN THE PUBLIC HEALTH SYSTEM**  Definition: The structures, functions, and accountabilities of specific organizations within the public health system. Where are they located within government and non-government structures and the larger health system? How is the non-government sector engaged? Whose role is it to generate public-health knowledge and use it for public health?  *Example:*   - *Organizations engaged with COVID-19 response in government (centers for disease control, universities, hospitals, labs)* - *Internal hierarchies, roles, and accountability mechanisms within key organizations and relations among them* - *Organizations charged with health emergency response and more general emergency response* \| \| **GOVERNANCE**  Definition: Processes of decision-making leading to policies and implementation of actions that enable the government to carry out its objectives.  *Example:*   - *Parliamentary procedures or bureaucratic routines that influence who participated and in what roles for key decision points* - *Emergency procedures to manage a crisis* - *How scientific and other forms of evidence are introduced and used in critical decisions* - *Political actors influencing critical decisions carried out by public-health organizations and others* \| |
| --- | --- | --- | --- | --- |
|  |
| **Additional file 2:** Scoping review protocol (abbreviated) |

**The purpose** of this scoping review is to synthesize and characterize literature to date on the impact of ‘upstream’ institutional, political, organizational, and governance factors in pandemic response. In particular, this scoping review will explore how these terms (institutions, politics, organizations, and governance) have been defined and operationalized in relation to epidemic and pandemic preparedness and response.

**Search Strategy:** The following four databases will be searched, with the intention of retrieving records from multiple disciplinary perspectives (e.g. medicine, social sciences, grey literature): JSTOR, PAIS, Web of Science, and Ovid Medline. A sample search strategy is as follows:

- (Institution* OR legal OR enforc* OR organi?ation OR politc* OR ideolog* OR elect* OR policy OR decision OR sociopolitical OR govern* OR regulation* OR regulatory)

**AND**

- (Pandemic* OR "infectious disease event" OR epidemic*)

**AND**

- (“state of emergenc*” OR “declaration adj4 emergenc*” OR “public health crisis” OR “public health emergenc*” OR “state adj4 emergenc*”)

**NOT**

- (opioid OR opinion OR pre-pandemic OR survey)

No date or source restrictions will be applied to the search, with the exception of a few databases (e.g., JSTOR, PAIS) with capabilities limited to searching journal articles, magazines, and reports.

**Screening:** Database results will be exported as .ris files and imported into *Covidence©* for two-stage eligibility screening by two independent reviewers (Level 1: Title/Abstract; Level 2: Full Text). Eligibility criteria for article inclusion will be as follows:

1. Does the article discuss at least one IPOG factor (institutions, politics, organizations, or governance) in relation to decision-making or policy development for public health crisis preparedness and/or response?
2. Is the article in English?

Reviewers will discuss all conflicts until consensus as to article inclusion or exclusion is reached.

**Data Extraction and Analysis:** Details as to data extraction and analysis will be determined once screening is complete. A sample data extraction table may be as follows:

| **Article (authors/year/title)** | **Study Location** | **Disciplinary Lens(es)** | **Methodology (Quant/Qual/Mixed)** | **IPOG factors defined and used** | **Definition/Operationalization of IPOG term(s) in Study** |
| --- | --- | --- | --- | --- | --- |
| *e.g. Smith et al. (2020).* The role of institutions in shaping COVID-19 decision-making in the UK | UK/Europe | Political science | Qualitative | • Institutions (I)  • Politics P) | •Institutions defined/applied as...  • Politics defined/applied as... |

Analysis is likely to include basic descriptive statistics (e.g. proportions of articles in relation to use/definition of IPOG terms, study methodology, location, etc.) and thematic analysis within and across articles with respect to how IPOG factors have reportedly influenced decision-making in prior research, as well as the framing/application of these concepts in relation to public health crisis response.

| **Additional file 3:** Developing an organizational map (organogram) |
| --- |

**The purpose** of the organizational map will be to visually represent the key actors and organizations involved in BC’s COVID-19 emergency response and the relationships between them (e.g. of authority/reporting, coordination). These relationships may be laid out in formal mechanisms such as legislation and regulations as well as in less formal mechanisms such as Memorandums of Understanding or scopes/statements of work. Our objectives for this procedure as as follows:

1. **Hierarchy of the public health structure:** To understand how the organizations within the public health system are structured in terms of vertical, horizontal, and other linkages across organizational units.
2. **Key actors/organizations:** To identify governmental, non-governmental, public and private organizations/bodies pertinent to the emergency response within BC’s public health system.
3. **Relationships among key actors/organizations:** To understand how key actors or organizations are connected, what connects them, and some key elements of how they function in relation to each other.
4. **Identify key informants to be interviewed:** To highlight pertinent public health organizations and to identify key informants holding specific roles within these organizations that were part of the decision-making processes during the COVID-19 emergency in BC.

**Procedure**: Our approach will be modified from the [WHO’s Tool for mapping governance for health and wellbeing: an organigraph method](https://www.euro.who.int/__data/assets/pdf_file/0011/389999/20181218-h1015-toolkit.pdf) and will involve the following steps:

1. Decide on the **context/focus** of the organogram (e.g. pre-COVID, early 2020 response)
2. Start by exploring **publicly available data**, specifically governmental health websites, to identify existing actors/organizations. We will focus on organizations directly involved in the emergency response. We will keep track of individuals/organizations and/or their roles in an MS Excel or Word document.
3. Draw a **“skeleton” organogram** starting to denote hierarchy among organizational units with information found from governmental websites, using MS PowerPoint.
4. **Broaden the search scope** by including academic journals, governmental action plans and/or other relevant documents that include organizational models, to add detail.
5. **Choose relationships to describe:** Develop a system of ‘shapes and connectors’ (and accompany legend) to denote types of organizational units (e.g. government, public institutions, non-government associations) and connecting relationships (e.g. ‘advising’, ‘informing’, ‘enforcing’).
6. **Create a document that visually describes the public health organizational structure:** Each organizational unit/role will be represented in one text box, connected to others with lines/arrows that describe the type of relationship between them.

We will document gaps in our understanding of the organizational structure and relationships, and will use the key informant interviews as opportunities to validate our mapping and make changes/additions, as necessary.

| **Additional file 4:** Creating an epidemic curve and timeline of associated ‘decision clusters’ in the BC government’s response |
| --- |

**The purpose** of the timeline is to describe and document the epidemiological progression of COVID-19 in British Columbia, Canada, including key ‘decision clusters’ that can be explored further in key informant interviews. This procedure will involve generating a database of public health and social measures (PHSMs) employed and plotting them over an epidemiological curve detailing the number of COVID-19 cases and deaths in BC on a weekly or monthly basis.

We conceptualize key moments in the progression of the COVID-19 pandemic as ‘clusters’ of decisions, rather than individual decision points. These **‘decision clusters’** might include the rollout of multiple public health and social measures that share a similar date and theme, such as restrictions on multiple types of gatherings, as well as financial supports for businesses, organizations, and individuals. For example, the Declaration of a Provincial State of Emergency in British Columbia, Canada in March 2020 included a series of associated decisions related to gathering restrictions and initial closures that were enacted into early April 2020.

**Data collection**: Data for the timeline will be found through a variety of academic and non-academic sources, including government websites, reports, policy monitors and media. In British Columbia, both the Government of BC News page and media have tracked PHSM during the pandemic. Other sources may include the *North American COVID-19 Policy Response Monitor* and the European Observatory on Health Systems and Policies Response *“COVID-19 Health System Response Monitor”*. The World Health Organization has tracked and compiled these measures into one dataset that is also publicly available. Other sources include *Our World in Data* (Oxford) and *Johns Hopkins Coronavirus Resource Center*.

**We propose the following steps for creating the timeline:**

1. Use case and mortality data to create an epidemiological curve. Compile the case and mortality data into a spreadsheet (e.g., using Excel) and create a graph based on either weekly or monthly case and death counts. This graph will be used as the foundation for the timeline graphic. We may convert daily counts into weekly or monthly counts by making a PivotTable, after which the PivotTable can be used to create a bar chart to visualize the epidemiological progression of the pandemic.
2. Create a second spreadsheet listing BC’s PHSMs from which the key ‘decision clusters’ will be determined, including public health interventions as well as a wide variety of responses across government ministries, such as social measures. We will use the WHO definition of non-pharmaceutical interventions (NPI), which includes “*any publicly announced program, statement, enforceable order, initiative or operational change originating from any government body in response to COVID-19 - whether to curtail its transmission or mitigate its social and economic ramifications.*”
3. Create a document (e.g., using PowerPoint) that visually demonstrates the case and mortality counts, along with key ‘decision clusters’ and pandemic ‘phases’ along the timeline (represented in textboxes with lines connecting these ‘clusters’ and ‘phases’ to their coinciding time periods).

From the visualization of events (‘decision clusters’ and ‘phases’) in relation to epidemiological data, we may identify questions to ask in key informant interviews.

| **Additional file 5:** Semi-Structured Interview Guide |
| --- |

**Background/Context:**

1. Please describe your professional background and titles and positions (in which organizations) relevant to the COVID-19 pandemic response in the period leading up to March 18^th^ 2020.
2. How did you come to serve in those positions? (e.g. how were you appointed and recruited and by whom?) To whom did you report in those positions?

**Activities leading up to March 18^th^, 2020 declaration of a provincial public health emergency:**

1. At what point did you realize the seriousness of the new virus? What evidence led you to realize it was serious?
2. When did you first hear about discussions in BC considering declaring a state of emergency? Please describe how you were informed about that and how it related to your role and position at the time.
3. Were you personally involved in discussions about when and how to declare a state of emergency in BC? In what ways were you involved?
4. What information was used to make the decision? What was the reasoning behind the decision?
5. Who else was involved in the decision-making procedure? What were their roles and positions?
6. Were there other processes that you heard about or were informed about but not participate in personally? Please describe.
7. What were your perceptions about the role or impact of federal-level decisions on these processes?

**Activities following the March 18th declaration:**

1. How did your role and work change as a result of the declaration? For example, did the organization employing you change? Did your supervision or reporting relationships change? Did your direct supervisory role change? Were you given any new tasks and resources?
2. Several specific orders were launched under the authority of the declaration. For example, schools were closed as were businesses with liquor licenses. In the next few days other businesses were closed and border closings occurred. What was your role in relation to these orders? With whom did you work or collaborate? What influenced those decisions?

**Roles/participation in other key decisions after March 2020 (ask participant to identify):**

1. How did your role and position change during this time?
2. What was your role specifically in relation to this decision(s)? Please describe in some detail regarding people you worked with and your observations about the decision processes. How were these different from the earlier period? What information informed these decisions?
3. Were you involved in some way with efforts in January to develop a test for the virus? If so, please describe how you were involved.

**Overall perception of process:**

1. Considering these different decision points in the evolution of the COVID-19 pandemic in BC, how would you describe your main engagement or contribution?
2. In your view, how was scientific evidence heard and attended to (if at all)?
3. In your engagement or contributions during that time, were you directly meeting with or communicating with persons holding political office, such as elected officials? Please describe some examples.
